# Supplementary material for: Effectiveness of Physical-Literacy-Based Online Education on Indices of Physical Fitness in High-School Adolescents: Intervention Study during the COVID-19 Pandemic Period
Source: Children (Basel). 2023 Oct 8;10(10):1666. doi: 10.3390/children10101666 (PMC10605366; doi:10.3390/children10101666)
Supplement: Supplementary file 1 [file children-10-01666-s001.zip › children-2638892-supplementary.pdf]

Supplementary file

Original educational videos in Croatian language with English subtitles are available here: https://pl-hl-nl.kifst.hr/pl/pl-int/
